# Decreased serum exosomal miR‐29a expression and its clinical significance in papillary thyroid carcinoma

**DOI:** 10.1002/jcla.23560

**Published:** 2020-12-23

**Authors:** Qiuting Wen, Yulou Wang, Xingjiang Li, Xiangguo Jin, Guimei Wang

**Affiliations:** ^1^ Department of Pathology Qiqihar Medical University Qiqihar China; ^2^ Department of General Surgery The First Affiliated Hospital of Qiqihar Medical University Qiqihar China

**Keywords:** biomarker, miR‐29a, papillary thyroid carcinoma, serum exosomes

## Abstract

**Background:**

Aberrant levels of circulating microRNAs (miRNAs) are potential biomarkers in papillary thyroid carcinoma (PTC) diagnosis and therapy. The aim of this study was to evaluate serum exosomal miR‐29a expression as a non‐invasive biomarker for PTC diagnosis and prognosis.

**Methods:**

Quantitative reverse transcription polymerase chain reaction was applied to measure serum exosomal miR‐29a expression levels in blood samples of 119 patients with PTC and 100 control subjects.

**Results:**

Serum exosomal miR‐29a expression levels were significantly decreased in PTC cases. In addition, receiver operating characteristic (ROC) analysis revealed serum exosomal miR‐29a could well differentiate PTC from normal controls. Moreover, serum exosomal miR‐29a levels increased progressively and significantly 30 days and 90 days after surgery. Furthermore, PTC patients with lower serum exosomal miR‐29a expression had higher risk of recurrence. Decreased serum exosomal miR‐29a expression was significantly associated with worse clinical variables including tumor size, extrathyroidal extension, and TNM stage, as well as shorter survival. Finally, both univariate and multivariate identified serum exosomal miR‐29a as an independent prognostic indicator for overall survival.

**Conclusion:**

These results demonstrated that serum exosomal miR‐29a might serve as a potential biomarker for PTC diagnosis and prognosis.

## Graphical abstract

The association between serum exosomal miR‐29a level and recurrence of PTC.

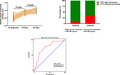

## 1 INTRODUCTION

Thyroid cancer is the most frequent tumor of the endocrine system, and its incidence rate gradually increases around the world.^1^ Thyroid cancer can primarily be divided into papillary, follicular, medullary, and anaplastic subtype. Papillary thyroid carcinoma (PTC) is the most common subtype of thyroid cancer and accounts for about 80% of all thyroid cancer cases.^2^, ^3^ Generally, the prognosis is relatively good for most of patients with PTC. However, the recurrence rate and mortality rate are still unfavorable for advanced‐stage patients with PTC, especially for those with aggressive phenotypes.^4^, ^5^ Therefore, more studies are required to be conducted to discover promising biomarkers of PTC for its early detection, effective therapy, and prognosis evaluation.

MicroRNAs (miRNAs) are a class of short (19‐25 nt) non‐coding RNAs that regulate the expression of target genes through either mRNA degradation or translational repression.^6^ Studies have demonstrated that miRNAs involve in diverse biological processes, including cell proliferation, metabolism, differentiation, and apoptosis.^7^ miRNAs can function as either oncogenes or tumor suppressor genes, depending on their specific gene targets.^8^ For instance, the expression of miR‐98‐5p was reduced in PTC. Knockdown of miR‐98‐5p promoted the malignant activities of PTC cells, and vice versa. These findings indicate that miR‐98‐5p plays a tumor suppressive role in PTC.^9^ Exosomes are small membrane‐enclosed extracellular vesicles of approximately 30‐100 nm and widely distributed in the blood, urine, and other bodily fluids.^10^, ^11^ Increasing evidence has revealed that most of serum‐circulating miRNAs are enriched in exosomes and can be stably detected.^12^ Therefore, serum exosomal miRNAs may be useful biomarkers for cancer diagnosis and prognosis prediction.

In terms of PTC, miR‐29a has been reported to play a role in the invasion and metastasis of this malignancy. Wang and colleagues showed that miR‐29a was dramatically reduced in PTC tissues and cells. Restoration of miR‐29a significantly attenuated cancer cell proliferation, invasion, migration in vitro, and tumor growth in vivo by regulating DPP4.^13^ Likewise, a reduction in miR‐29a expression was observed in PTC tissues and its downregulation was strongly associated with worse clinical features. miR‐29a upregulation or AKT3 inhibition greatly restrained carcinogenesis and metastasis of PTC in vitro and in vivo.^14^ However, the clinical significance of serum exosomal miR‐29a as a potential biomarker for PTC has not yet explored. In this study, we investigated whether serum exosomal miR‐29a expression was associated with the clinical outcome of patients with PTC.

## 2 MATERIALS AND METHODS

### 2.1 Patients and serum

The study was approved by the Ethics Committee of Qiqihar Medical University. All examinations were carried out after obtaining written informed consent from patients and healthy volunteers. In this study, a total of 119 patients with PTC, comprising 57 men and 62 women, who underwent surgical resection in our hospital were enrolled. Patient parameters, including age, gender, histological subtype, tumor location, lymph node metastasis, tumor size, extrathyroidal extension, and TNM stage, were retrospectively collected (Table 1). Tumor stages were determined using the classification guidelines of the American Joint Committee on Cancer (AJCC). Blood samples were collected from 119 PTC subjects and 100 healthy volunteers as controls. Additionally, paired blood samples were obtained from all patients with PTC at 30 days and 90 days after the operation. All samples were centrifuged at 1200 *g* at 4°C for 10 minutes. The supernatants were then divided into small aliquots and stored at −80°C until use.

**Table 1:** Association between clinical factors and serum exosomal miR‐29a expression

Variables	Number of patients	High serum exosomal miR‐29a	Low serum exosomal miR‐29a	*P*
Age (years)				
<50	71	33	38	.7693
≥50	48	21	27	
Gender				
Male	57	25	32	.7497
Female	62	29	33	
Tumor location				
Unilateral	63	33	30	.1036
Bilateral	56	21	35	
Lymph node metastasis				
No	34	20	14	.0624
Yes	85	34	51	
Tumor size (cm)				
<2	78	41	37	.0299
≥2	41	13	28	
Extrathyroidal extension				
No	94	49	45	.0041
Yes	25	5	20	
TNM stage				
Ⅰ/Ⅱ	78	44	34	.0009
Ⅲ/Ⅳ	41	10	31	

### 2.2 Cell culture

The PTC cell lines (PTC‐1, BCPAP) and the normal control cell line Nthy‐ori3‐1 were cultured in RPMI‐1640 (Invitrogen) supplemented with 10% fetal bovine serum, 100 U/mL penicillin, and 1 μg/mL streptomycin. All cell lines were maintained in a humidified incubator at 37°C and 5% CO_2_.

### 2.3 Exosome isolation

Exosome isolation from serum samples was performed using ExoQuick Exosome Precipitation Solution (System Biosciences) according to the manufacturer's protocol. Serum samples were thawed and centrifuged at 3000 *g* for 15 minutes to remove cell debris. ExoQuick Exosome Precipitation Solution (SBI System Biosciences) was added to the supernatant, and the mixture was incubated at 4°C, followed by centrifugation at 1500 *g* for 30 minites. The exosome pellets were re‐suspended in PBS and stored at –80°C until use. Similar procedures were performed to isolate the exosomes from the culture media.

### 2.4 Exosomal RNA isolation and quantitative reverse transcription PCR

For exosomal RNA extraction, total RNA was extracted from serum samples and culture media using a Qiagen miRNeasy Mini kit (Qiagen). The quality of total RNAs was determined using an Agilent 2100 Bioanalyzer (Agilent Technologies). The reverse transcription reaction was performed using the TaqMan MicroRNA Reverse Transcription Kit (Applied Biosystems) according to the manufacturer's instructions. The quantitative reverse transcription PCR (qRT‐PCR) reactions were run on a 7500 Real‐Time PCR System (Applied Biosystems). All reactions were performed in triplicate. The relative serum exosomal miR‐29a expression levels were calculated using the 2^−ΔΔ^ *^C^* ^t^ method, and spiked‐in Cel‐miR‐39 was used as a normalizer for serum samples.

### 2.5 Western Blot

The proteins were separated by 10% SDS‐PAGE and transferred to a nitrocellulose membrane (Santa Cruz Biotechnology). After blocking with 5% skimmed milk for 1 hour at room temperature, membranes were incubated with anti‐TSG101 (Santa Cruz Biotechnology) and anti‐CD63 (Abcam) overnight at 4°C. Then, the membranes were incubated with secondary antibodies for 1 hour at room temperature. The signal was visualized using a Western Blotting Luminol Reagent (Santa Cruz Biotechnology).

### 2.6 Statistical analysis

Statistical analyses were performed with GraphPad Prism 8.0 (GraphPad Software, La Jolla, California, USA) or MedCalc v12 (MedCalc software, Belgium). The Mann‐Whitney *U*‐test was used to analyze the difference between two groups. The correlations between serum exosomal miR‐29a and the clinicopathological factors were assessed with the chi‐squared test. Receiver operating characteristic (ROC) curves and the area under the ROC curve (AUC) were used to analyze the feasibility of serum exosomal miR‐29a as a diagnostic tool for PTC. Overall survival (OS) and relapse‐free survival (RFS) curves were constructed using the Kaplan‐Meier method, and differences were calculated using Log‐rank tests. The independent prognostic factors for PTC were identified using univariate and multivariate Cox proportional hazards regression analysis. *P* < .05 was considered statistically significant.

## 3 RESULTS

### 3.1 Serum exosomal miR‐29a expression in PTC patients and its diagnostic value

**Figure 1:**
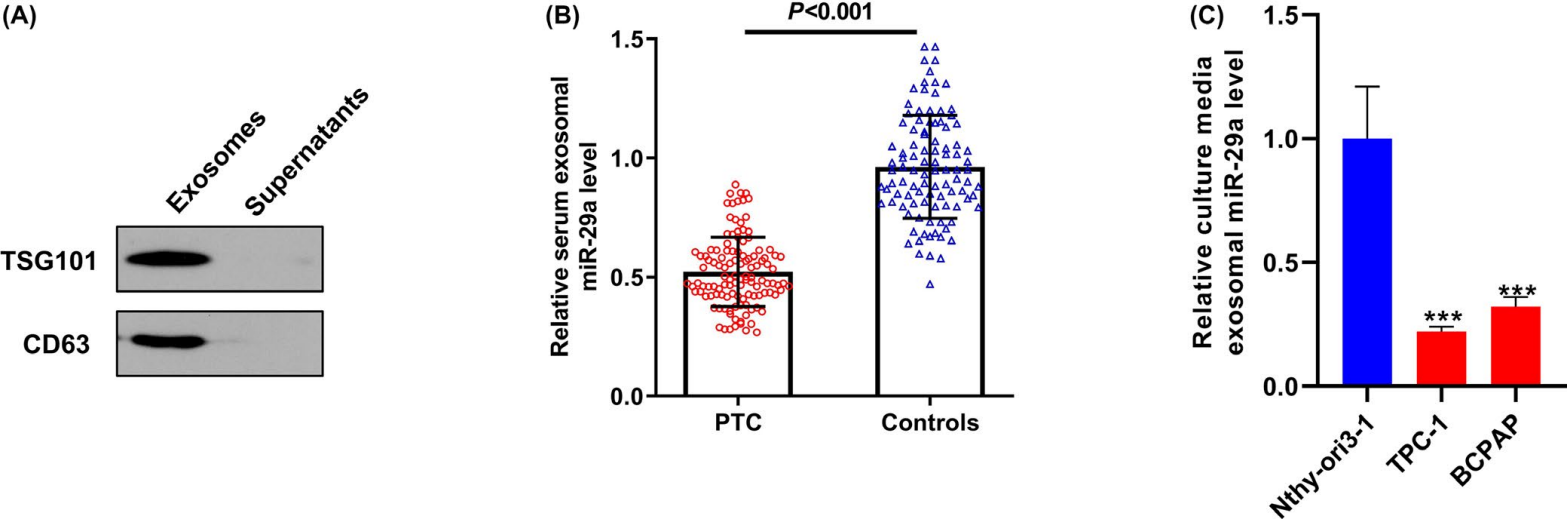
A, The exosomes isolated from the serum samples were positive for exosomal biomarkers TSG101 and CD63. B, Serum exosomal miR‐29a levels in papillary thyroid carcinoma (PTC) cases were significantly lower than those in controls. C, The culture media exosomal miR‐29a level was significantly lower in PTC cell lines than in the control cell line

The Western blot results demonstrated that the exosomes we isolated from the serum samples were positive for the exosomal biomarkers TSG101 and CD63 (Figure 1A). Then, the expression levels of serum exosomal miR‐29a were detected in 119 patients with PTC and 100 healthy controls using qRT‐PCR. As shown in Figure 1B, serum exosomal miR‐29a levels in PTC subjects were markedly downregulated compared with those in healthy controls (*P* < .001). Interestingly, we have compared the level of exosomal miR‐29a in the culture media from PTC cell lines and (TPC‐1 and BCPAP) the normal control cell line Nthy‐ori3‐1. Our results showed that the culture media exosomal miR‐29a level was significantly lower in PTC cell lines than in the control cell line (*P* < .001) (Figure 1C), which further support our findings that serum exosomal miR‐29a level was remarkably reduced in patients with PTC compared with the healthy individuals.

**Figure 2:**
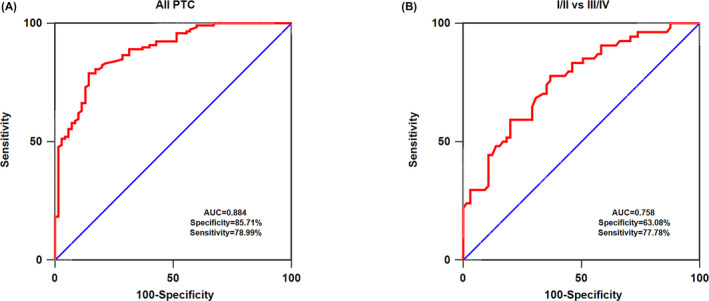
A, receiver operating characteristic (ROC) curve to assess the diagnostic accuracy of serum exosomal miR‐29a level in all papillary thyroid carcinoma (PTC) patients compared with controls. B, ROC curve to assess the diagnostic accuracy of serum exosomal miR‐29a level in I/II stage PTC patients compared with III/IV stage PTC patients

Receiver operating characteristic analysis was applied to evaluate the diagnostic efficiency of serum exosomal miR‐29a in differentiating PTC cases from healthy individuals. As presented in Figure 2A, serum exosomal miR‐29a exhibited AUC value of 0.884 in the discrimination of PTC from normal controls, with a specificity of 85.71% and sensitivity of 78.99%. Also, serum exosomal miR‐29a demonstrated a decreased AUC value to 0.758, 77.78% sensitivity, and 63.08% specificity in distinguishing early‐stage patients with PTC from advanced‐stage patients with PTC (Figure 2B).

### 3.2 Increased serum exosomal miR‐29a levels in PTC patients after surgery

**Figure 3:**
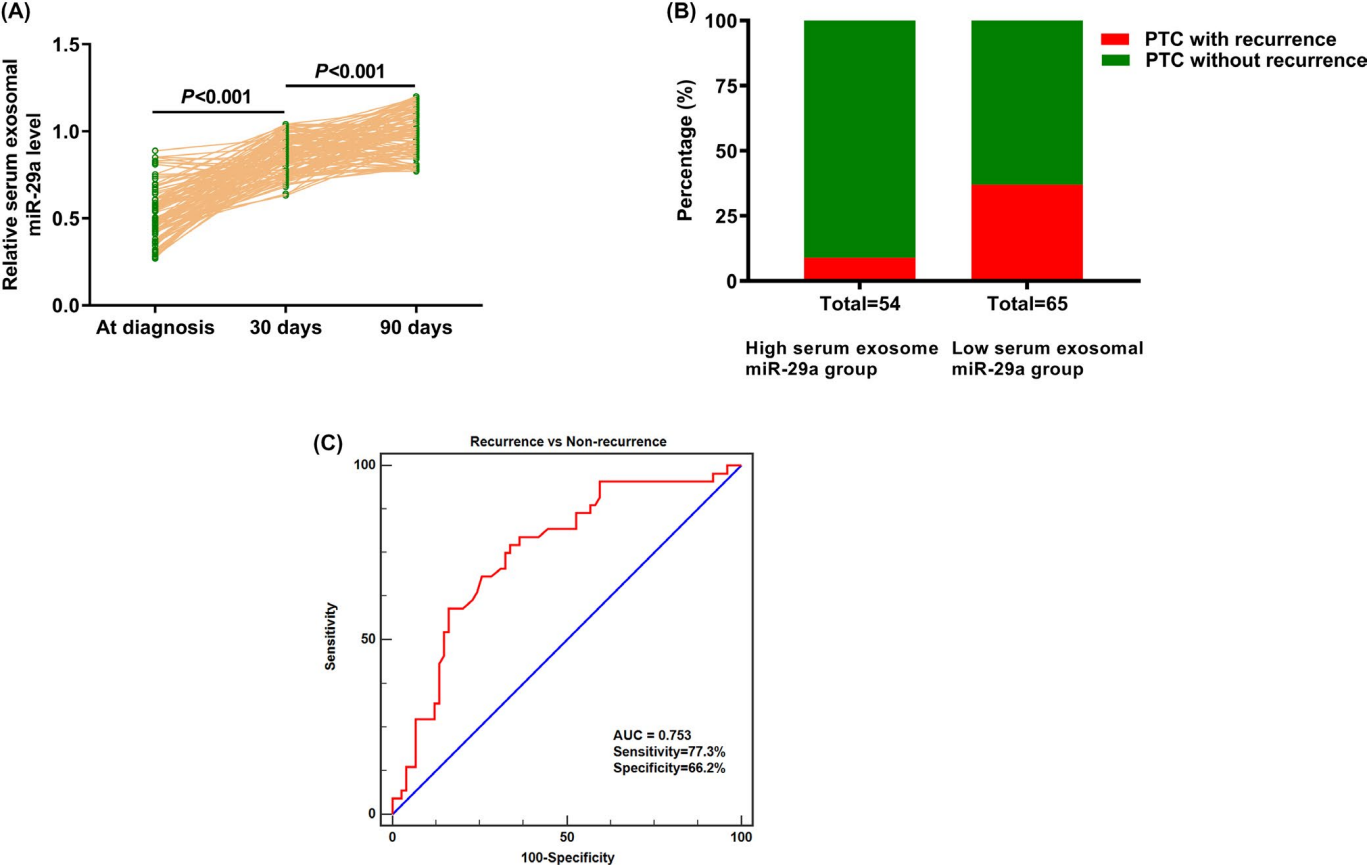
A, Serum exosomal miR‐29a expression levels were progressively and significantly increased at 30‐ and 90‐d following surgery. B, papillary thyroid carcinoma (PTC) patients with lower serum exosomal miR‐29a levels were more prone to develop a recurrence. C, The AUC value of serum exosomal miR‐29a for discriminating recurrence cases from non‐recurrence cases was 0.753. The sensitivity and specificity were 77.3% and 66.2%, respectively

Next, we detected serum exosomal miR‐29a levels in patients with PTC at 30 days, 90 days following surgery. Serum exosomal miR‐29a levels in blood samples collected 30 days after surgery were significantly higher than those in blood samples collected before surgery (*P* < .001, Figure 3A), and serum exosomal miR‐29a levels in blood samples collected 90 days after surgery were significantly elevated compared with those collected 30 days after surgery (*P* < .001 Figure 3A). Thus, serum exosomal miR‐29a expression levels were gradually and significantly increased at 30‐ and 90‐days following surgery and could be useful for monitoring treatment response and postoperative progression.

It is important that 5 of 54 (9.2%) PTC subjects in high serum exosomal miR‐29a expression group were diagnosed with recurrence after surgery, and 25 of 65 (38.5%) PTC cases in low serum exosomal miR‐29a group were confirmed to have recurrence after surgery, suggesting that PTC patients with lower serum exosomal miR‐29a levels had a higher risk of recurrence (Figure 3B). The AUC value of serum exosomal miR‐29a for discriminating recurrence cases from non‐recurrence cases was 0.753. The sensitivity and specificity were 77.3% and 66.2%, respectively (Figure 3C).

### 3.3 Serum exosomal miR‐29a expression and clinicopathological features

The association between serum exosomal miR‐29a expression and clinicopathological parameters was then assessed. Patients with PTC expressing serum exosomal miR‐29a at levels above the median level were assigned to the high expression group (n = 54), and those cases with expression less than the median value were assigned to the low expression group (n = 65). As shown in Table 1, serum exosomal miR‐29a downregulation was strongly correlated with tumor size (*P* = .0299), extrathyroidal extension (*P* = .0041), and TNM stage (*P* = .0009). Whereas, no significant association was found between serum exosomal miR‐29a expression and age (*P* = .7693), gender (*P* = .7497), tumor location (*P* = .1036), and lymph node metastasis (*P* = .0624).

### 3.4 Decreased serum exosomal miR‐29a expression predicted poor prognosis of PTC patients

**Figure 4:**
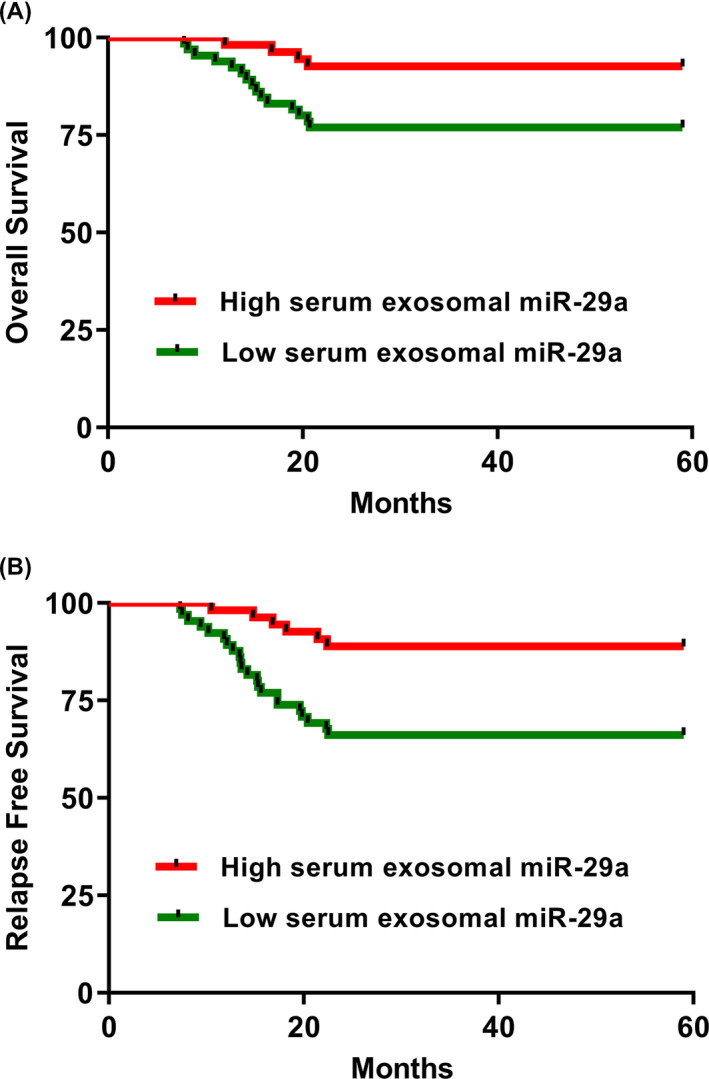
A, Kaplan‐Meier curve for overall survival in papillary thyroid carcinoma (PTC) patients stratified by serum exosomal miR‐29a expression. B, Kaplan‐Meier curve for relapse‐free survival in PTC patients stratified by serum exosomal miR‐29a expression

To analyze the correlation of serum exosomal miR‐29a with prognosis of patients with PTC, the Kaplan‐Meier method was used to plot OS and RFS curves. As shown in Figure 4A, the OS rate was significantly higher in PTC patients with high serum exosomal miR‐29a expression in comparison with those with low serum exosomal miR‐29a expression (*P* = .0189). Likewise, the RFS rate in PTC patients with low serum exosomal miR‐29a expression was significantly lower compared with those with high serum exosomal miR‐29a expression (*P* = .0030, Figure 4B). Using univariate analysis of the Cox regression model, four prognostic factors were identified to have statistical significance: serum exosomal miR‐29a (RR = 3.52, 95% CI = 1.68‐5.63, *P* = .014), tumor size (RR = 2.40, 95% CI = 1.31‐3.68, *P* = .022), extrathyroidal invasion (RR = 2.93, 95% CI = 1.54‐4.71, *P* = .017), and TNM stage (RR = 4.56, 95% CI = 1.95‐7.32, *P* = .004). In addition, multivariate analysis showed these four prognostic factors were also independent prognostic markers for OS of PTC: serum exosomal miR‐29a (RR = 3.85, 95% CI = 1.76‐6.25, *P* = .010), tumor size (RR = 2.78, 95% CI = 1.43‐4.27, *P* = .020), extrathyroidal invasion (RR = 3.34, 95% CI = 1.62‐5.23, *P* = .015), and TNM stage (RR = 4.81, 95% CI = 2.09‐7.86, *P* < .001) (Table 2).

**Table 2:** Univariate and multivariate analyses of the prognostic factors for overall survival in all papillary thyroid carcinoma patients

Parameters	Univariate analysis			Multivariate analysis		
	RR	95% CI	*P*	RR	95% CI	*P*
Serum exosomal miR‐29a	3.52	1.68‐5.63	.014	3.85	1.76‐6.25	.010
Tumor size	2.40	1.31‐3.68	.022	2.78	1.43‐4.27	.020
Extrathyroidal extension	2.93	1.54‐4.71	.017	3.34	1.62‐5.23	.015
TNM stage	4.56	1.95‐7.32	.004	4.81	2.09‐7.86	<.001

## 4 DISCUSSION

Screening for the early detection of PTC was crucial to improve patient survival and facilitate cancer prevention. In this study, serum exosomal miR‐29a levels in PTC cases were significantly decreased compared with those in controls. In addition, serum exosomal miR‐29a was a promising biomarker for screening patients PTC from normal controls and early‐stage PTC from advanced‐stage PTC. Moreover, serum exosomal miR‐29a levels in PTC cases at 90 days following surgery were significantly higher than those in PTC cases at 30 days following surgery, or in patients with PTC before surgery. Furthermore, low serum exosomal miR‐29a expression was closely associated with aggressive clinical parameters, shorter OS/RFS and identified as an independent prognostic biomarker in patients with PTC. The data suggested that serum exosomal miR‐29a might function as a tumor suppressor in PTC.

miR‐29a had also been reported as a tumor suppressor by regulating the expression of cancer‐related target genes in some malignancies. In gastric cancer (GC), miR‐29a expression was markedly downregulated in GC tissues and enforced miR‐29a expression significantly suppressed cell proliferation and induced cell cycle arrest through silencing p42.3 or VEGF‐A expression.^15^, ^16^ Xiong et al found that miR‐29a was frequently underexpressed in melanoma cell lines. miR‐29a upregulation inhibited cancer cell viability and growth in vitro by inversely regulating Bmi1.^17^ In addition, a significant decrease in miR‐29a expression was found in prostate cancer patient samples. Ectopic miR‐29a expression remarkably suppressed carcinogenesis by degrading KDM5B expression.^18^ In lung cancer, miR‐29a expression levels were significantly downregulated in cancerous tissues in comparison with normal tissues. miR‐29a overexpression repressed the oncogenic activities of cancer cells and increased chemosensitivity of cancer cells to cisplatin by targeting NRAS.^19^ miR‐29a downregulation was not only detected in glioblastoma but also in glioma, and miR‐29a overexpression dramatically inhibited cancer cell proliferation, migration, and invasion and stimulated cell apoptosis by targeting TRAF4 or QKI‐6.^20^, ^21^

In contrast, several studies have reported that miR‐29a was overexpressed in some cancer types. For example, Deng and colleagues revealed that miR‐29a upregulation occurred more frequently in cholangiocarcinoma (CCA) tissues, and high miR‐29a expression was associated with worse clinical variables of CCA and shorter survival.^22^ In breast cancer (BC), Wu et al showed that miR‐29a expression was highly expressed in BC tissues. In vitro and in vivo analysis demonstrated that miR‐29a upregulation or SUV420H2 inhibition significantly stimulated carcinogenesis of BC.^23^ Similarly, Pei et al found that enforced miR‐29a expression dramatically increased cell proliferation, migration of BC cell lines, and miR‐29a knockdown exhibited the opposite effects on cell proliferation and invasion.^24^ Moreover, Chen et al demonstrated that miR‐29a overexpression was closely correlated with metastasis of hepatocellular carcinoma (HCC) and worse clinical outcomes.^25^ Sun et al^26^ showed that an inverse correlation between miR‐29a and TTP expression was found in pancreatic cancer tissues and cell lines. Ectopic miR‐29a expression significantly displayed a significant oncogenic effect in vitro and in vivo. Furthermore, Qiu et al^27^ showed that miR‐29a upregulation not only enhanced cell migration and invasion of nasopharyngeal carcinoma (NPC), but also predicted shorter overall survival of patients with NPC. Tang et al^28^ showed that miR‐29a overexpression was highly correlated with poor prognosis in patients with colorectal cancer. Upregulation of miR‐29a significantly stimulated cancer cell invasion through degrading KLF4. Interestingly, the circulating level of miR‐29a was found to be significantly increased in patients with lung cancer,^29^ which was contradictory to the findings in the lung cancer tissues.^19^ One possible explanation for the different expression of miR‐29a in different types of cancers or even in the same cancer type is that the concrete role of miR‐29a in tumorigenesis might depend on the specific tumor microenvironment and the downstream targets in regulates.

We have revealed that serum exosomal miR‐29a levels were significantly lower in patients with PTC than in healthy controls. Low serum exosomal miR‐29a expression was associated with worse prognosis. Taken together, serum exosomal miR‐29a was a reliable novel biomarker of PTC diagnosis and prognosis.
